# A Three-dimensional Comparison of Pre- and Post-component Position in a Series of Off-label Robotic-assisted Revision Total Knee Arthroplasties

**DOI:** 10.1016/j.artd.2023.101310

**Published:** 2023-12-27

**Authors:** Micah MacAskill, Richard Peluso, Jonathan Lash, Timothy E. Hewett, Matthew Bullock, Alexander Caughran

**Affiliations:** Department of Orthopaedics, Marshall University Joan C. Edwards School of Medicine, Huntington, WV, USA

**Keywords:** Robotic assisted, Revision, Knee replacement, Sagittal alignment, Condylar offset

## Abstract

**Background:**

The application of robotic-assisted arthroplasty in revision knee scenarios continues to evolve. This study compares the pre- and post-revision implant positions in series of revision total knee arthroplasties (TKA) using a robotic arm system.

**Methods:**

Twenty-five consecutive off-label robotic-assisted revision TKA were performed. After virtual revision femoral and tibial components were positioned to achieve “balanced” medial and lateral flexion and extension gaps, the existing primary implants (PI) were removed, and bone cuts were executed with the robotic arm system. Preoperative coronal, sagittal, and axial position of the PI was compared to the final planned positions of the robotic revision implants (RRI) for each subject. A repeated measures ANOVA using the absolute difference in millimeters and degrees between the PI and RRI orientation was completed.

**Results:**

Intra-operatively, the virtual gaps were balanced within the planning software followed by successful execution of the plan. There was a statistically significant difference between posterior condylar offset and tibial component positioning for RRI compared to PI. There was no difference between the distal femoral component values between PI and RRI.

**Conclusions:**

The sagittal alignment of the revision implants, specifically the femoral posterior condylar offset and tibial component slope, are statistically significant considerations for a stable revision TKA with off-label use of a robotic-arm system. Other potential benefits may include appropriate implant sizing which can affect the resultant ligamentous tension important for a functional revision TKA. Future research and software iterations will be needed to determine the overall accuracy and utility of robotic-assisted revision TKA.

## Introduction

Knee arthroplasty alleviates pain and restores function for nearly 900,000 Americans every year, and by 2030, this number is expected to more than double because the aging population desires to remain active [1]. As the number of expected primary knee arthroplasties continues to grow, so will revision procedures. The most recent report from the American Joint Replacement Registry (2022) has indicated infection, aseptic loosening, mechanical complications, and instability comprise over 80% of the causes of knee revision surgery in the United States [2]. The sum of technical errors during the index procedure can significantly affect postoperative function. For instance, the removal of additional bone from the distal femur, over-resection of the posterior condyles, and addition of slope to the tibia result in gap asymmetry which contributes to prosthesis instability. In response, implant companies have attempted to standardize primary total knee arthroplasty (TKA) via robotic-assisted surgery, but the application of robotic-assisted surgery in revision scenarios is still largely uncharted. It can be difficult to adapt current technology to revision scenarios because of bone loss, ligament attenuation, and a lack of appropriate computer software.

Current implant systems rely on intramedullary (IM) fixation to bypass areas of deficient bone during revision knee arthroplasty. Smaller fully cemented stems can be used without much difficulty, but longer diaphyseal engaging IM stems have been found to influence the final position of the revision implants [[3], [4], [5]]. Anatomically speaking, it can be difficult to precisely position a long-stem revision tibial component because the center of the tibial epiphysis is not colinear with the center of the diaphysis. Similarly, when a long stem of the revision femoral component contacts the sagittal bow of the femur, disruption of gap balance may occur [6,7]. Therefore, implant augmentation and offset couplers were developed to improve bone contact and fine tune implant positioning. Unfortunately, offset components only permit a few millimeters of fine tuning within the plane of resection yet can continue to inhibit ideal placement of revision implants. Frequently, a constrained polyethylene component is employed during revision TKAs to supplement for residual instability after revision implants are placed.

Revision components can be positioned independently of IM stems during revision TKA by using a previously described technique [8,9]. By manipulation of a current robotic-assisted workflow, the authors completed a series of robotic-assisted revision TKAs to address a variety of patients presenting with infection, aseptic loosening, mechanical complications, and instability. The purpose of this study was to compare the 3-dimensional changes in the preoperative and postoperative positions of the knee arthroplasty components necessary to achieve balanced gaps within a patient cohort. It is hypothesized that posterior femoral condylar offset is more important to gap stability than distal femoral measurements in robotic revision TKAs.

## Material and methods

Institutional review board approval was obtained prior to the initiation of this study. A total of 25 consecutive cases by 2 fellowship-trained arthroplasty surgeons at a single intuition from July 2021 through March 2023 were reviewed. All subjects had undergone a prior TKA using conventional jig-based instrumentation. Patients with a prior unicompartmental knee arthroplasty or peri-prosthetic fracture were excluded. Patient demographics along with indications for revision can be found in Table 1.

**Table 1:** Patient demographics.

Patient	Age (years)/sex	Laterality	Primary implants	Reason for revision
1	66 M	Right	Biomet Vanguard PS	Aseptic loosening
2	63 M	Right	Conformis PS	Instability
3	84 F	Right	Depuy Attune PS	Instability
4	64 F	Right	Smith & Nephew Journey PS	Instability
5	60 F	Right	Stryker Triathlon CR	Instability
6	70 F	Left	Depuy Sigma PS	Aseptic loosening
7	69 F	Right	Biomet Vanguard PS	Aseptic loosening
8	65 M	Left	Depuy Sigma PS	Aseptic loosening
9	73 M	Right	Zimmer Persona CR	Instability
10	55 F	Left	Zimmer NextGen PS	Instability
11	69 M	Right	Articulating Spacer	Infection
12	62 F	Right	Smith & Nephew Legion CR	Instability
13	80 M	Left	Smith & Nephew Legion CR	Instability
14	72 F	Right	Biomet Vanguard CR	Instability
15	66 M	Right	Zimmer Persona PS	Aseptic loosening
16	72 M	Left	Depuy Attune PS	Aseptic loosening
17	53 F	Right	Articulating Spacer	Infection
18	68 M	Right	Smith & Nephew Journey CR	Instability
19	65 F	Left	Biomet Vanguard PS	Instability
20	50 M	Left	Smith & Nephew Journey PS	Instability
21	70 F	Right	Zimmer NextGen CR	Instability
22	71 F	Left	Biomet Vanguard CR	Instability
23	59 F	Right	Articulating Spacer	Infection
24	59 M	Left	Articulating Spacer	Infection
25	62 M	Right	Depuy Attune CR	Aseptic loosening

All surgeries were performed using the MAKO robotic-arm system (Stryker Orthopedics, Mahwah, NJ) equipped with Version 1.0 software according to our previously described surgical technique [8,9]. In most cases, several attempts were necessary to ensure an overall registration error of ≤0.5 mm due to a metal artifact from the existing implants on the preoperative computed tomography (CT) scan. Anterior referencing was used for all cases due to posterior femoral bone loss and to avoid anterior femoral notching. The revision femoral and tibial components were manipulated within the coronal, sagittal, and axial planes to obtain balanced medial and lateral flexion and extension gaps (Table 2). After careful implant removal, all bone cuts were performed with the robotic arm, and implant augmentation was utilized in each case depending on the requirements for gap balancing. Preparation for the IM stem and metaphyseal cone(s) was performed for the femur and tibia as necessary. For most cases, the tibial array was positioned within the tibial diaphysis while the femoral array was located at the distal femur metadiaphyseal region to ensure room for a revision construct with short IM stems.

**Table 2:** Revision implant sizes and gap measurements.

Patient	Initial sizes (Femur/Tibia)	Balanced sizes (Femur/Tibia)	Gaps (extension/Flexion)	Augments (Femur/Tibia)	Metaphyseal cone (Femur/Tibia)	IM stem (Femur/Tibia)	Polyethylene
1	6/7	5/6	29 M; 29 L/28 M; 29 L	5 DM; 15 DL; 10 PM; 10 PL/10	1&2/A	15 × 150/12 × 50	11^a^
2	7/8	7/7	25 M; 26 L/25 M; 25 L	10 DM; 10 DL; 10 PM; 10 PL/5	-/D	19 × 150/12 × 50	9^a^
3	4/3	3/3	21 M; 21 L/19 M; 19 L	5 DM; 5 DL; 5 PM; 5 PL/-	-/A	15 × 100/12 × 50	13^b^
4	5/5	6/5	19 M; 19 L/19 M; 19 L	5 DM; 5 DL; 5 PM; 10 PL/5	1&2/A	12 × 100/15 × 50	11^b^
5	2/3	3/3	22 M; 22 L/22 M; 22 L	5 DM; 5 DL;5 PM; 5 PL/-	-/A	14 × 150/12 × 50	16^a^
6	6/5	5/4	22 M; 22 L/22 M; 22 L	5 DM; 5 DL/5	-/A	12 × 100/12 × 50	19^a^
7	3/3	3/2	20 M; 20 L/20 M; 20 L	15 DM; 10 DL; 10 PM; 10 PL/10	-/B	13 × 100/12 × 50	9^a^
8	6/6	5/5	19 M; 19 L/18 M; 19 L	5 DM; 5 DL; 5 PM; 5 PL/-	-/A	19 × 100/12 × 50	13^a^
9	8/8	7/6	19 M; 18 L/21 M; 21 L	10 DM; 10 DL; 5 PM/-	-/A	22 × 100/12 × 50	9^b^
10	4/4	3/3	18 M; 18 L/19 M; 19 L	5 DM; 5 DL; 5 PM; 5 PL/-	-/A	13 × 100/12 × 50	16^a^
11	5/6	7/6	28 M; 28 L/28 M; 28 L	5 DM; 10 PM; 10 PL/10	-/A	-/12 × 50	11^b^
12	1/2	2/3	18 M; 19 L/18 M; 22 L	5 DM; 5 DL; 10 PM; 10 PL/-	-/A	12 × 50/12 × 50	11^a^
13	8/8	8/7	19 M; 19 L/19 M; 19 L	5 DM; 10 PM; 10 PL/-	-/A	12 × 50/12 × 50	11^a^
14	2/3	4/3	24 M; 23 L/29 M; 28 L	5 DM; 15 DL; 10 PM; 10 PL/10	-/A	12 × 100/12 × 50	11^a^
15	6/6	7/6	25 M; 25 L/25 M; 24 L	5 DM; 5 DL; 5 PM; 5 PL/5	-/-	15 × 50/12 × 50	9^a^
16	7/6	6/5	19 M; 19 L/19 M; 19 L	5 DM; 5 DL; 10 PM; 10 PL/5	-/A	19 × 150/12 × 100	11^a^
17	3/3	2/2	18 M; 19 L/18 M; 18 L	5 DM; 5 DL/10	-/-	12 × 100/15 × 50	13^a^
18	4/3	4/3	17 M; 18 L/18 M; 18 L	5 PM; 5 PL/5	-/A	12 × 100/12 × 50	11^a^
19	4/3	4/4	33 M; 33 L/33 M; 33 L	15 DM; 15 DL; 10 PM; 10 PL/10	-/B	20 × 100/12 × 50	16^a^
20	5/5	4/4	18 M; 19 L/18 M; 19 L	5 PM; 5 PL/-	-/A	12 × 100/12 × 50	11^b^
21	2/2	2/3	20 M; 16 L/25 M; 20 L	5 DM; 5 DL; 10 PM; 10 PL/-	-/A	14 × 100/12 × 50	11^a^
22	3/3	5/5	23 M; 24 L/22 M; 23 L	10 DM; 5 DL; 10 PM; 5 PL/5	-/A	15 × 100/12 × 50	11^a^
23	5/4	6/5	33 M; 33 L/40 M; 40 L	15 DM; 15 DL; 10 PM; 10 PL/10	-/C	20 × 100/15 × 50	13^a^
24	5/5	5/4	19 M; 19 L/19 M; 19 L	10 PM; 10 PL/-	-/A	9 × 100/12 × 100	13^a^
25	4/5	7/6	19 M; 19 L/12 M; 19 L	5 DM; 5 DL; 10 PM; 10 PL/5 M	-/C	-/12 × 50	11^a^

M, MEDIALl L, lateral; D, distal; P, posterior; DM, distal medial; DL, distal lateral; PM, posterior medial; PL, posterior lateral.

a Constrained.

b Posterior-stabilized.

For each subject, the preoperative coronal, sagittal, and axial positions of the primary implants (PI) were compared to the final coronal, sagittal, and axial positions of the robotic revision implants (RRIs). The current MAKO computer software (Version 1.0) cannot directly measure existing implant position nor directly calculate the positional difference between PI and RRI; therefore, an indirect method was utilized for this study.

**Figure 1:**
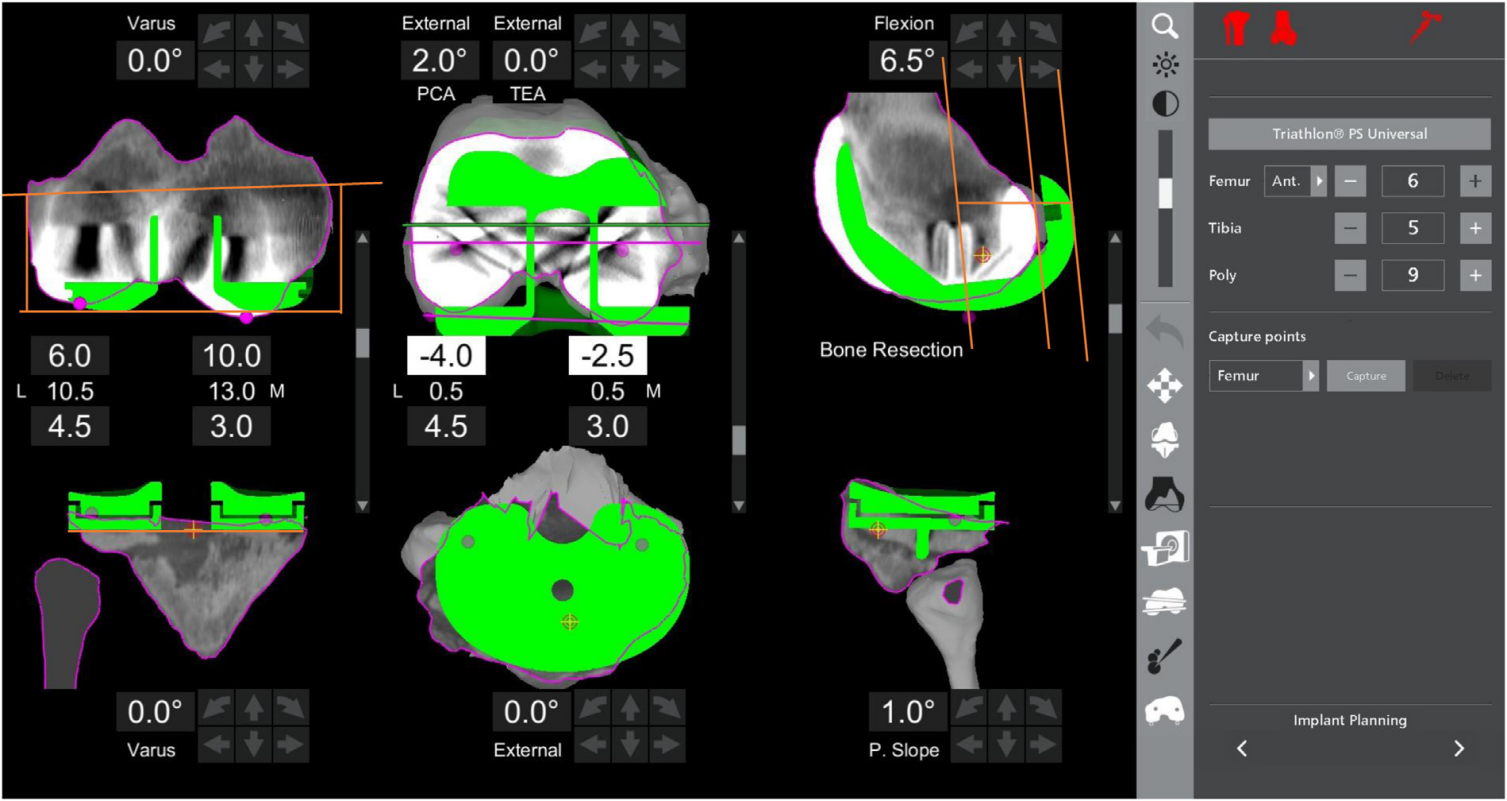
Robotic implant planning page depicting the final position of the robotic revision implant (RRI) in green overlying the Primary implants (PI) in white. Top right: Note the increased posterior offset between the RRI and PI necessary to balance the revision TKA. Metal artifact can obscure bony landmarks, but by viewing multiple slices of the CT scan, we can best estimate the location of the bony landmarks. Because revision TKA is not yet indicated for the MAKO robotic system, revision implants and intramedullary stems are not currently available for virtual templating.

After a specific CT scan of the operative extremity was obtained, the PI was outlined during the implant planning phase. Next, virtual trial implants were superimposed over the existent PI to determine preoperative implant position within the cardinal planes (Fig. 1). The best-fit virtual implants were centered within areas of metal artifact to obtain the most accurate measurement possible as indicated by prior studies [10,11]. The following values were recorded in relation to the defined mechanical axis or transepicondylar axis (TEA) of the extremity: PI femoral component flexion/extension, varus/valgus, internal/external rotation, posterior condylar axis, and TEA and PI tibial component varus/valgus, slope, and internal/external rotation.

After the robotic assisted revision surgery was completed, the final planned virtual positions of the RRI were analyzed, and the following values were recorded in relation to the defined mechanical axis or TEA of the extremity: RRI femoral component flexion/extension, varus/valgus, internal/external rotation positions, posterior condylar axis, and TEA and RRI tibial component varus/valgus, slope, and internal/external rotation.

In order to obtain data on the resultant change in component positioning, screenshots of the implant planning page that depicted the final RRI that overlayed the preoperative CT scan with the PI were then transferred into TraumaCad (Brainlab Munich, Germany). Measurements from the medial and lateral epicondyles to the distal most portion of the medial and lateral condyles for the PI and RRI were recorded, respectively. The difference between these numbers yielded the net change between PI and RRI positions for the distal medial and distal lateral condyles (Tables 3 and 4). Measurements for posterior condylar offset (PCO) for the medial and lateral condyles were also obtained by measurement of the distance from a line tangential to the posterior femoral shaft and apex of the posterior medial and posterior lateral condyles for the PI and RRI. The difference between these values demonstrated the net change between PI and RRI positions for the posterior medial and distal lateral condyles, respectively (Fig. 1). Calibration of the screenshots was standardized from a known constant value of either the inner width of the femoral implant box (16.2 mm) or the medial-lateral width of the tibial baseplate (which varies by size). All data were secured within a password-protected spreadsheet.

**Table 3:** Implant measurements from Bony landmarks (in millimeters).

Patient	ME to PI	ME to RRI	LE to PI	LE to RRI	PC to medial PI	PC to medial RRI	PC to lateral PI	PC to lateral RRI
1	32.1	33.8	25.4	23.6	22.7	26.1	18.8	18.4
2	31.7	31.2	38.3	37.8	22.4	23.4	24.8	27.3
3	36	37	27.6	29.1	19.3	23.2	22.8	24.8
4	39.6	40.6	26	30.2	22.2	26.2	22.4	26.3
5	33.9	34.9	31.2	31.7	22.4	21.9	23.4	24
6	31.1	34.9	32.1	37.6	18.7	24.1	24.5	25.3
7	17	29	22.9	24.4	19.2	21.1	23	27.2
8	26	27.2	30.7	29.9	19.4	24.1	25.9	29.9
9	35.8	40.1	32.8	34.9	29	26.3	26.9	25.1
10	26.6	27	28.3	26.8	17.6	18.6	24.3	27
11	40.5	40.3	31	30.5	24.7	29.1	22.4	30.7
12	33.4	34.1	27.1	28.5	19.3	19.7	20.2	23.4
13	45.6	40.5	37	38.1	21.3	29.3	20	25.9
14	24.2	28.7	22.5	26.2	27.7	27.1	25.3	26.7
15	36.8	36.9	26.3	26.5	26.3	22.5	23.3	25.1
16	35.8	32	39.4	36.6	21.1	26.3	19.2	22.3
17	32.1	26.5	22.4	21.6	25.7	19.3	23	18.8
18	34.1	27.7	26.6	20.3	18.8	21.6	20.7	24.8
19	22.1	23.4	22.8	23.7	20	16.1	20.7	19.6
20	27.5	23.4	30.4	28.6	15.6	21.9	21.1	24.3
21	26	30.1	22.3	21.1	14.1	20.5	17.9	22.8
22	33.1	31.4	24.5	25.4	17.5	18	26.6	27
23	36.3	34.9	28.6	31.5	15.4	21.3	19.2	26.7
24	29.5	29.8	38.8	37.7	18.7	23.6	20.7	25.1
25	37.2	36.9	26.7	26.2	19.5	28.8	20.9	24.7

ME, medial epicondyle; LE, lateral epicondyle; PC, posterior condyle; PI, primary implant; RRI, robotic revision implant.

**Table 4:** Implant measurements (in degrees).

Patient	PI femoral flexion	RRI femoral flexion	PI femoral varus/valgus	RRI femoral varus/valgus	PI tibial varus/valgus	RRI tibial varus/valgus	PI tibial slope	RRI tibial slope	PI PCA	RRI PCA	PI TEA	RRI TEA
1	−2.6	3.9	5.1	−0.4	0	0	9	2	0.9	−2.6	−2.5	−6
2	11.5	5.4	−1.5	−2.5	2.4	1.9	8.5	3	8.1	2.6	8.4	2.9
3	0.5	9	−2.5	−1	2	0	−0.5	1	−1.2	−2.2	1.1	0.2
4	0.5	5.5	0	0	−0.5	0	8.5	1	0.1	0.1	0	0
5	0.5	8	0	0	−3.5	0	3	3	1.6	1.6	0	0
6	1.5	1.5	−1.5	0	0	0	3.5	0	0.6	−5.9	6.5	0
7	4.5	6	12.5	−0.9	0	0	0	2	4.5	1.5	9.4	7.9
8	3.5	3.5	3.5	0.5	7	0	4.5	0	−1.1	−1.1	0	0
9	−0.5	6	3.7	0.2	5	0	7	2	−0.6	0.9	1	2.5
10	3	4	2.6	−0.4	−1	0	8.5	3	0.4	1.9	5.5	7
11	6.5	6.5	0.5	0.4	0	0	5.5	2.5	2.1	4.6	−0.6	1.9
12	1.5	6	0.5	1.3	3	0	14	3	1.3	3.3	2.9	4.9
13	10.5	8.5	−3.5	2.7	1.5	−3	−0.5	0	2	−1.5	1.1	−2.4
14	−3	6	−0.1	−0.1	1	0	2	2	0.8	2.2	0.4	1.9
15	16.5	6	−0.7	0.4	1.5	0	2.5	3	1	5.1	1.1	5
16	5	8	−0.2	0.1	15	0	−5.5	0	−0.3	−2.3	2.5	0.5
17	6	6	−4.7	1.2	6.5	0	6	0	−1.1	2.9	−1.5	1.9
18	9	8	0	0.5	3	3	−3	0	1.8	2.3	3.5	4
19	3.5	3.5	1.5	0.5	−1	0	1	3	0.2	0.2	0.1	0.2
20	2.5	5.5	−1.2	−0.1	0.5	0	−1	1	4.5	−4	5.5	−3
21	17	7.5	7	0.4	0.5	0	6	1	1.3	−2.8	5.2	3
22	9	6	−1.5	0.4	3.5	0	4.5	0	0.4	0	4.9	4.2
23	3	6.5	−4.5	0	2.5	0	13.5	1	2	0	0.2	2
24	10	8.1	1.6	1.7	8.5	−1	3	2.5	1.4	−1.2	0.3	−2.3
25	5.5	4	1.4	0	14	0	13	3	0.8	−6.2	6.9	0

Femoral flexion: (+) flexion (−) extension; varus/valgus: (+) varus (−) valgus; posterior condylar axis (PCA): (+) external rotation (−) internal rotation; transepicondylar axis (TEA): (+) external rotation (−) internal rotation; tibial slope (+) posterior slope (−) anterior slope.

Repeated-measures analysis of variance (ANOVA) using the difference (in millimeters and degrees preoperatively and postoperatively) were used to determine if there were statistical differences between the final positions of the RRI and the PI. Following that determination, multiple ANOVAs between the PI and RRI absolute values in millimeters and degrees were conducted to compare differences between groups before and after the robotic revision TKA surgeries. Differences were considered statistically significant at *P* < .05.

## Results

### Statistical determinations

There were statistically significant differences between the placement of the RRI and the PI. The overall repeated-measures ANOVA comparing the difference between primary and revision implants (in millimeters) demonstrated that the RRI values were statistically significantly different from the PI (*P* < .001). There were also statistically significant differences in the absolute values (in millimeters) for both the posteromedial and posterolateral condyles from PI to RRI (*P* values). The average increase in posterior medial and lateral condyle offset was 4.2 mm and 3.3 mm, respectively. There was no statistically significant difference between the distal medial or distal lateral values between PI and RRI. The posterolateral and posteromedial offset difference between implants (in mm) was statistically significantly different from the PI (*P* < .001 and *P* < .03 respectively). While the difference between the distal-lateral and distal-medial positioning of the implants (in mm) was not significantly different from the PI (*P* = .82 and *P* = .87, respectively).

Preoperative and postoperative measured absolute values (degrees) in the tibial component positioning, but not femoral component positioning, were different between the RRI and the PI. The tibial slope and varus/valgus values were statistically different from the PI; however, the femoral values flexion, varus/valgus, and posterior condylar axis were not significantly different from one another. The difference of tibial varus/valgus between implants (in degrees) was statistically significantly different from the PI (*P* < .003). The posterior-medial distance to implant (in mm) was statistically significantly different from the PI (*P* < .03). The RRI tibial slope postoperative vs preoperative (degrees) was also statistically significantly different from the PI (*P* < .01).

The femoral posterior condylar axis in the RRI (in degrees) was not statistically significantly different from the PI placement although it approached significance with *P* = .0988. Femoral flexion in the RRI procedure (in degrees) was not statistically significantly different from the PI (*P* = .51). Femoral varus/valgus in the RRI procedure (in degrees) was not statistically significantly different from the PI placement (*P* = .68). The RRI surgical TEA (degrees) was not statistically significantly different from the PI placement (*P* = .285).

A subcategory ANOVA comparing the PI and RRI implant positions among subjects with aseptic loosening, infection, and instability did not reach statistical significance although this study was not powered for subgroup analysis given the current sample size.

### Implant results

There were no reported complications during the operative procedure (ie, fracture, neurovascular injury, ligamentous injury, or anesthesia concerns) or during the postoperative hospitalization. For all cases, the selected tibial component was within one size over/under the selected femoral component per manufacturer recommendations. Medial and lateral flexion and extension gaps were found to be within 1 mm of each other in most cases.

Tibial metaphyseal cones were used more often than femoral cones. Twenty-three of the revision tibias utilized a short 12 × 50-mm IM stem. A revision femoral component was utilized in 23 of the cases. The diameter of the femoral IM stem varied by case, with the average length being 100 mm. Offset couplers were not utilized for any of the cases. The average polyethylene thickness employed during revision cases was 11 mm. Twenty cases utilized a constrained posterior-stabilized component while 5 cases utilized a regular posterior stabilized component.

Medial and lateral tibial augments were used 60% of the time. One case required a step cut on the tibia, and therefore only used a single 5-mm medial tibial augment. During femoral augmentation, 5-mm augments were more commonly used distally while 10-mm augments were more commonly used posteriorly.

The existing femoral component preoperative coronal plane alignment varied between 4.7° valgus and 12.5° varus while the existing tibial component preoperative alignment was found between 3.5° valgus to 14° varus. The average postoperative coronal implant positioning averaged 0.20° varus and 0.04° varus for the femoral and tibial components, respectively.

The postoperative posterior condylar and trans-epicondylar axes averaged 0.24° internal rotation and 1.45° external rotation, respectively, which was slightly less than the preoperative average values of 1.26° external rotation and 2.47° external rotation, respectively.

The preoperative femoral component flexion ranged from 2.6° of extension to 16.5° of flexion. Final femoral implant flexion averaged 5.9°, and this was closely monitored to balance the flexion gap during implant planning. Postoperative posterior tibial slope averaged 1.6° in this cohort.

## Discussion

Restoration of femoral PCO and near-neutral alignment of the tibial component are statistically significant parameters necessary to achieve more symmetric gaps in this revision TKA cohort. Review of the PI positions from the current cohort frequently revealed a larger distal femoral cut and overresection of the posterior femoral condyles during the index surgeries. The resultant instability arises from the subtle elevation of the joint line and decreased PCO of the femoral component leading to decreased ligamentous balance [5,[12], [13], [14]]. The ensuing flexion instability was further amplified when excess posterior slope was added to the primary tibial component.

Robotic-assisted revision TKA simplifies several challenging concepts frequently encountered during revision scenarios. Knowledge of each patient’s mechanical axis can provide the surgeon with a frame of reference when orienting the femoral and tibial components to help address gap symmetry. Conventional revision TKA relies on an IM alignment rod to indirectly reference the femoral mechanical axis. This traditional method is based on “average” measurements that can vary based on patient sex, age, height, and body mass index [15,16]. During our study, the mechanical axis of the operative extremity was directly obtained from a preoperative CT scan, which represented ideal neutral alignment during surgery.

Incorporating ligamentous gap values during virtual implant templating was preferred to standard preoperative radiographic templating. Results of this study indicated one-third of the radiographically templated femoral components were oversized while another one-third were found to be undersized when compared to the final implanted femoral component (Table 2). This can be partially attributed to magnification error seen with radiograph templating. Alternatively, a different femoral size can be selected to alter the ligamentous tension to achieve gap symmetry.

To the authors’ knowledge, there are no “defined” ligamentous tension values for revision TKA representing a void within TKA literature. The referenced “gap” values within our results serve as a proxy for the gap measurements during robotic revision TKA. Incorporating ligamentous tension values throughout the range of motion may enhance the surgeon’s ability to optimally position the implants, which has the potential to improve gap symmetry in revision TKA, but further research is necessary.

Symmetric gaps both in flexion and extension as well as medially and laterally were the goals of the operative procedure, but it was difficult to achieve equal numbers in all cases. If this occurred, the priority was focused on a symmetric medial flexion and extension gap. This rationale is driven by the dynamic interplay of bony and soft-tissue balance within the medial compartment during normal knee mechanics [17].

The MAKO robotic arm system establishes boundaries for restricted kinematic alignment during the procedure where the implants are positioned within 3° to 5° of the mechanical axis within the coronal, sagittal, and axial planes [18]. Re-establishment of the joint line within 5 mm of the native location has been correlated with improved knee-joint function and satisfaction [12]. Joint line measurements were not obtained during this study because current robotic software (Version 1.0) cannot directly determine the joint line in a revision scenario. The authors conceptualize the joint line as a dynamic entity that is established by proper ligamentous tension. Indirect determination of a more native joint line may be possible by relying on kinematic collateral ligamentous tension throughout the knee range of motion [19].

Sagittal alignment is an underappreciated factor for stability and function during revision TKA [3,20,21]. Evaluation of the prosthesis sagittal implant position and ligamentous balance is difficult with manual instrumentation because of bone loss and soft-tissue laxity that are commonly encountered during revision scenarios [22]. PCO, a component of sagittal alignment, is an important measurement that has been studied for both primary and conventional revision TKAs. The PCO has been reported up to 40% greater than native measurements because of bone loss and soft-tissue attenuation [23]. PCO directly affects the flexion gap, but it can also indirectly affect the extension gap as theorized by Mitsuyasu et al [24]. The enlarged femoral condyles that result from increased PCO may stretch the remaining posterior knee structures when the knee assumes an extended position, thus contributing to extension gap stability [24].

**Figure 2:**
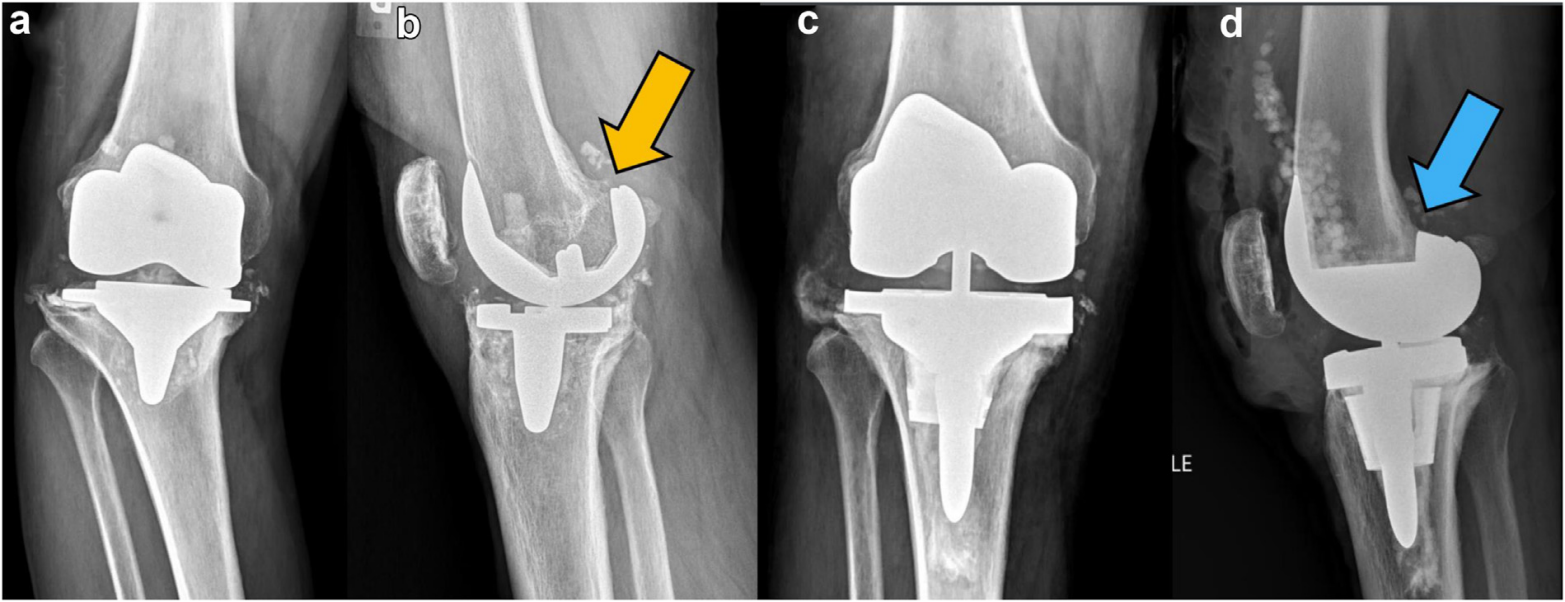
(a) Anteroposterior and (b) lateral preoperative radiographs of an unstable TKA with aseptic loosening of the tibial component that subsided into a varus position. The joint line was moved inferior during the index surgery. There is slight distalization of the femoral component (arrow) depicting a step-off at the posterior femoral condyles. (c) Anteroposterior and (d) lateral postoperative radiographs depict a balanced revision TKA with 5° flexion of the femoral component with increased posterior offset of the femoral component when compared to preoperative radiographs. The tibial component is now supported by a metaphyseal cone and a medial augment. Note the anterior flange rests on the anterior cortex, and the posterior aspect of the femoral component is confluent with the remaining femoral bone without step off (arrow). Mild varus obliquity of the joint line is characteristic of a kinematically aligned revision TKA [17]. The joint line has been indirectly restored based on the balanced ligamentous tension throughout the knee range of motion.

**Figure 3:**
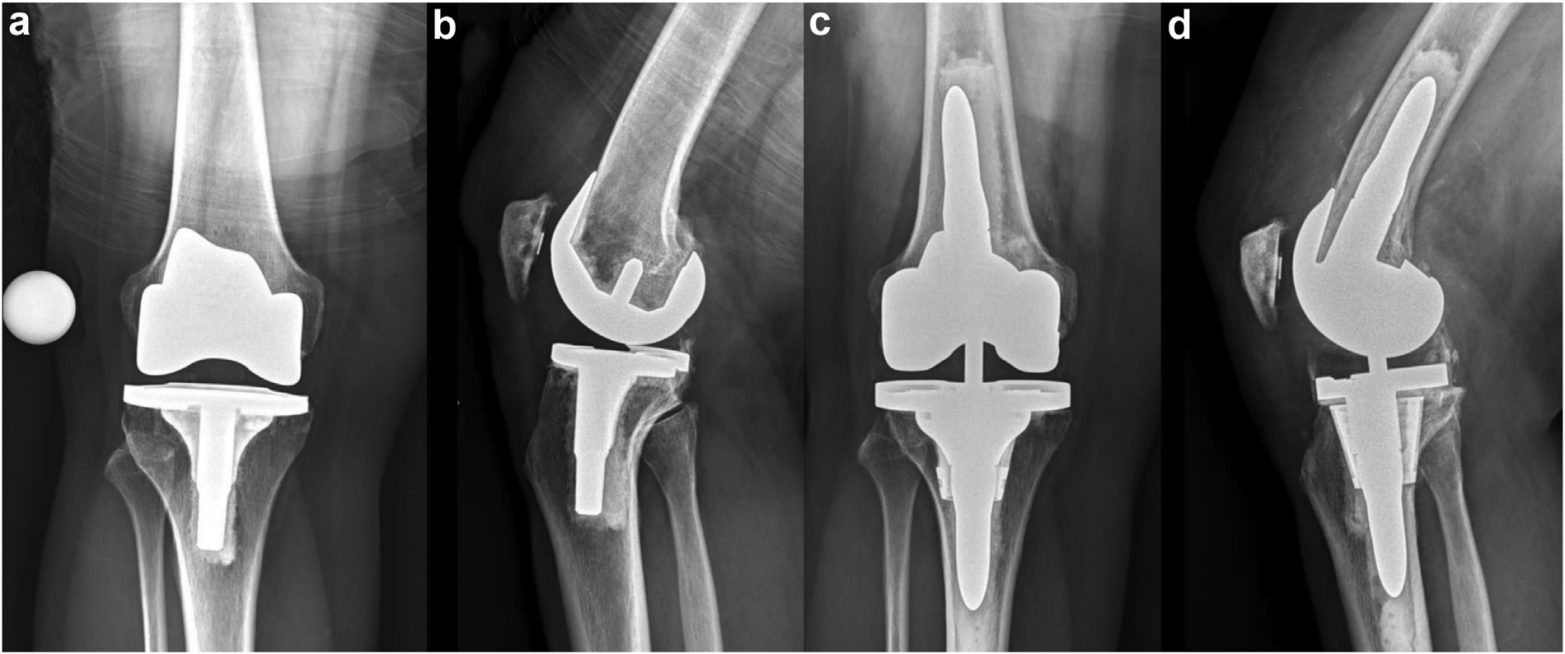
(a) Anteroposterior and (b) lateral preoperative radiographs of an unstable TKA. There is slight extension of the femoral component with overresection of the posterior femoral condyles and increased posterior slope on the tibial component, which contributed to the flexion instability in this patient. (c) Anteroposterior and (d) lateral postoperative radiographs depicting a balanced revision TKA with (3 degree) flexion of the femoral component and posterior femur augmentation to restore the lost femoral posterior condylar offset. Short revision IM stems with a tibial metaphyseal cone is characteristic of robotic assisted revision TKA.

A recent comparative study by Sultan et al [25] demonstrated robotic arm–assisted surgery more accurately restored PCO in primary TKA. Meanwhile Clement et al [26] reported PCO is an independent predictor of functional outcome after revision TKA. In addition, PCO directly correlates with patient satisfaction scores after revision TKA [26,27]. PCO along with a near-neutral tibial slope provides flexion stability and helps ensure proper ligamentous tension during mid and deep flexion [22,23,26]. For our revision TKA cohort, 3D virtual planning helped illustrate the associated changes in the sagittal alignment of the femoral and tibial components. In most cases, the revision femoral component was upsized, and the flexion orientation was optimized at the distal femur to help restore ligamentous tension and flexion gap symmetry during our revision procedures. The PCO was increased in 76% of the revision TKA in this study cohort. The medial PCO increased on average 4.2 mm while lateral PCO increased on average 3.3 mm. These medial and lateral values were not expected to be equal because of the resultant kinematic alignment for this revision TKA cohort. Within the present study, the posterior femoral condyles required larger augments than the distal femur to again address the larger flexion gap and maximize PCO (Figure 2, Figure 3).

With PCO being such a critical factor in revision TKA, the accuracy and precision of robotic-assisted surgery is of particular interest for future research. The accuracy of preoperative bone resections and final coronal limb alignment using the MAKO system has previously been reported to be within 1 mm of the plan and 0.78°, respectively [28]. Unfortunately, the accuracy of the robotic system during revision scenarios is still unknown.

Kang et al [29] reported that up to 7° of femoral component flexion increased the mechanical advantage of the quadriceps muscles and decreased the patellofemoral contact stress. On the contrary, excessive femoral component flexion can result in impingement between the femoral box and the polyethylene post during knee extension [21,29]. Between 1° and 2° of component flexion decreases the flexion gap by an average of 1 mm depending on implant geometry according to several studies [3,7,20,21]. For the current cohort, posterior translation of the femoral component occurred with femoral flexion beyond 5° in an attempt to keep the anterior flange flush with the anterior femoral cortex to prevent overstuffing the patellofemoral joint and disruption of extensor mechanism kinematics. The average femoral component flexion measured 5.9° for the current cohort, which was slightly larger than the 3° to 5° of flexion suggested during primary TKA [14]. The degree of femoral component flexion appears to be variable and dependent on patient sex and other anthropometric measurements including body height and weight in addition to gap balance [30,31].

Recent studies have shown that revision constructs that consist of cones and short stems can assist with improved implant alignment and reduced micromotion at the bone-implant interface [[32], [33], [34]]. In addition, it would be difficult to attain adequate femoral component flexion with an attached metaphyseal sleeve since they are directly coupled to the IM stem [4,33]. Furthermore, utilization of longer IM stems may cause the proximal tip to engage the anterior femoral cortex and inadvertently cause the femoral component to assume an extended position and disrupt gap balance [7,21,35].

Traditionally with conventional manual revision TKA, a series of trial-and-error scenarios are attempted to achieve “an acceptable” soft-tissue “feel” of a balanced knee. This method has been shown to be difficult to reproduce among surgeons because of a lack of objective data [36]. A dynamic interplay exists between the coronal, sagittal, and axial orientations of a knee prosthesis [37,38]. Adjustment of position within one plane can directly affect the other 2 planes due to implant geometry as calculated by the computer. Surgeons may now be able to further quantify these small incremental changes when using robotic assistance during revision procedures. Robotic revision TKA can provide the surgeon with immediate visual feedback regarding implant sizes and the need for augmentation. This information has the potential to improve operating efficiency and may limit the amount of time spent trialing during revision surgery.

This article demonstrates re-establishing the PCO contributes to intraoperative gap balance. Although beyond the scope of this study, the overwhelming majority of patients reported a more stable knee after their robotic revision surgery. Early in the process, the authors utilized fully constrained components for robotic revision cases due to the learning curve associated with the off-label use of the robotic arm system. Nonetheless, as the operative technique and experience evolved, the tendency now is to use a standard posterior stabilized component because of the ability to restore soft-tissue tension throughout knee range of motion as long as the collateral ligaments have not been compromised.

Finally, the ability to attain specific targets or achieve “optimal alignment” with robotic assistance does not always translate into clinical success and/or improved patient satisfaction because of the countless variables encountered during revision surgeries. Radiographs can depict ideal implant orientation and correlate with an impeccable physical examination, but in the end, the patient can still remain dissatisfied. Preoperative planning continues to be the gold standard of revision TKA surgery, but in the future, robotic assistance may permit intraoperative fine tuning of this plan, which may someday contribute to improved patient outcomes.

## Limitations

This study includes a cohort of 25 patients from a single institution. A prospective randomized controlled trial comparing manual to robotic revision TKA will be necessary to evaluate long-term outcomes of this technique. Future studies will need to include more patients across several institutions after a standardized technique is established. The accuracy of this robotic system in primary TKA has been reported, but application of the system during revision scenarios is still unknown [28]. Future studies may focus on quantifying changes in gap balance with the associated change in implant positions. This work is based on basic principles of restricted kinematic alignment referenced to a lower-limb mechanical axis, but other alignment philosophies exist. Currently there are no defined ligamentous tension values for knee arthroplasty; hence, attainment of a particular gap value may not fully represent physiologic performance that is applicable for all patients. Finally, the cost of robotic revision surgery is currently unknown. Forthcoming analyses incorporating costs from a preoperative CT scan, additional operating time, robotic system maintenance, software updates, disposable equipment, and correlation with patient outcomes are in order to establish the feasibility of robotic assistance for revision TKA.

## Conclusions

This current series demonstrates the authors’ up-to-date experience using the MAKO robotic system for revision TKA. Results of this study indicate that sagittal alignment of the revision implants, specifically the femoral PCO and tibial component slope, are statistically significant considerations for a stable revision TKA. By utilization of robotic assistance, the surgeon can become more cognizant of how appropriate implant size and alignment directly affects the ligamentous tension that is important for a functional revision TKA. The MAKO robotic arm system is not yet approved for revision scenarios, so the utility of this technique and outcomes of this study are still considered investigational. Future research and software iterations will be needed to determine the feasibility of robotic-assisted revision TKA.

## Conflicts of interest

The authors declare there are no conflicts of interest.

For full disclosure statements refer to https://doi.org/10.1016/j.artd.2023.101310.

## Author contributions

M.W.B., A.C., J.L., M.L.M., and R.P. contributed to writing—review & editing and writing—original draft. M.W.B. and A.C. contributed to supervision and framed the study methodology. M.W.B., A.C., and M.L.M. contributed to investigation and study conceptualization. T.E.H. performed formal analysis and data curation.

## CRediT authorship contribution statement

**Micah MacAskill:** Writing – review & editing, Writing – original draft, Investigation, Conceptualization. **Richard Peluso:** Writing – review & editing, Writing – original draft. **Jonathan Lash:** Writing – review & editing, Writing – original draft. **Timothy E. Hewett:** Formal analysis, Data curation. **Matthew Bullock:** Writing – review & editing, Writing – original draft, Supervision, Methodology, Investigation, Conceptualization. **Alexander Caughran:** Writing – review & editing, Writing – original draft, Supervision, Methodology, Investigation, Conceptualization.

## Appendix A Supplementary data

Conflict of Interest Statement for Bullock

Conflict of Interest Statement for Caughran

Conflict of Interest Statement for Hewett

Conflict of Interest Statement for Lash

Conflict of Interest Statement for MacAskill

Conflict of Interest Statement for Peluso
