# Supplementary material for: A Three-dimensional Comparison of Pre- and Post-component Position in a Series of Off-label Robotic-assisted Revision Total Knee Arthroplasties
Source: Arthroplast Today. 2023 Dec 27;25:101310. doi: 10.1016/j.artd.2023.101310 (PMC10788208; doi:10.1016/j.artd.2023.101310)
Supplement: Conflict of Interest Statement for Bullock [file mmc1.pdf]

# CONFLICT OF INTEREST STATEMENT

## *American Association of Hip and Knee Surgeons*

(Adopted from the American Academy of Orthopaedic Surgeons disclosure statement)

The following form **must be filled out completely and submitted by each author (example, 6 authors, 6 forms). All items require a response. If there is no relevant disclosure for a given item, enter "*None*."**

---

Manuscript Title

1. Royalties from a company or supplier (The following conflicts were disclosed)
**N/A**

2. Speakers bureau/paid presentations for a company or supplier (The following conflicts were disclosed)
**Smith & Nephew**

3A. Paid employee for a company or supplier (The following conflicts were disclosed)
**N/A**

3B. Paid consultant for a company or supplier (The following conflicts were disclosed)
**Smith & Nephew**

3C. Unpaid consultants for a company or supplier (The following conflicts were disclosed)
**N/A**

4. Stock or stock options in a company or supplier (The following conflicts were disclosed)
**Stryker - Smith & Nephew**

5. Research support from a company or supplier as a Principal Investigator (The following conflicts were disclosed)
**N/A**

6. Other financial or material support from a company or supplier (The following conflicts were disclosed)
**N/A**

7. Royalties, financial or material support from publishers (The following conflicts were disclosed)
**N/A**

8. Medical/Orthopaedic publications editorial/governing board (The following conflicts were disclosed)
**Editorial Board – Journal of Arthroplasty and Arthroplasty Today Journal**

9. Board member/committee appointments for a society (The following conflicts were disclosed)
**West Virginia Orthopaedic Society – AAHKS Digital Health and Social Media Committee**

**Each author must sign AND print or type his/her name, date and submit a separate form**

In addition, one BLINDED Conflict of Interest form (no author names used) should be submitted per manuscript with all author disclosures.

| Matthew Bullock DO | | 3/1/23 |
|---|---|---|
| Author Name (Print or Type) | Author Signature | Date |
